# Selective killing of circulating tumor cells prevents metastasis and extends survival

**DOI:** 10.1186/s13045-018-0658-5

**Published:** 2018-09-10

**Authors:** Yi Rang Kim, Jung Ki Yoo, Chang Wook Jeong, Jin Woo Choi

**Affiliations:** 1 Department of Hematology/Oncology, Yuseong Sun Hospital, Daejeon, 34084 Republic of Korea; 2 Department of Pharmacology, College of Pharmacy, Kyung Hee University, Seoul, 02447 Republic of Korea; 3 Department of Life and Nano-pharmaceutical Sciences, Kyung Hee University, Seoul, 02447 Republic of Korea; 4 Department of Urology, Seoul National University Hospital, Seoul, 03080 Republic of Korea

**Keywords:** Circulating tumor cells, Green fluorescent protein, Metastasis, Photodynamic therapy, Photosensitizers

## Abstract

Distant metastasis is initiated by circulating tumor cells (CTCs), which are considered to be a determining factor for the degree of metastasis and the survival of cancer patients. Although CTC-based diagnostic approaches are being rapidly developed, limited studies have proven the benefits of CTC elimination, with most studies providing only hypothetical inference because of the technical difficulty in examining the effects of CTC elimination in vivo. We modified photodynamic therapy to specifically eliminate green fluorescent protein (GFP)-expressing CTCs and evaluated the therapeutic efficacy of CTC elimination. When circulating blood is illuminated with a blue laser (*λ* = 473 nm), the combination of GFP and photosensitizers induces a selective elimination of GFP-expressing CTCs, with limited effect on normal cells. In GFP-expressing cancer cell-infused or transplanted mice models, the treatment suppressed distant metastasis and extended the survival of the tumor-bearing mice. Taken together, CTCs are a core seed to be metastasized into secondary organs and elimination of CTCs may improve the survival of cancer patients.

**Electronic supplementary material:**

The online version of this article (10.1186/s13045-018-0658-5) contains supplementary material, which is available to authorized users.

## ᅟ

Circulating tumor cells (CTCs) present in the vascular system are tumor cells that will metastasize from primary or disseminated tumors [1]. Rapid advancements in detection and isolation techniques have led to the remarkable discoveries on the role of CTCs and their association with cancer prognosis [2–7]. Since an increased number of CTCs are associated with poor prognosis, CTC-targeted therapies may provide a promising new approach which could improve cancer prognosis [8, 9]. However, the unpredictable nature and dynamics of CTCs and the lack of adequate treatment modalities hamper the selective targeting of CTCs.

**Fig. 1:**
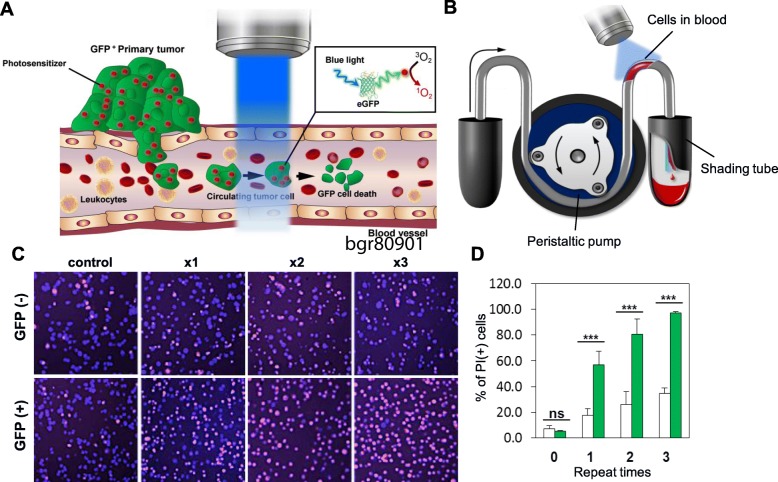
Concept of selective CTC-targeting PDT and in vitro blood circulation-mimicking system. **a** Scheme of the selective CTC-targeting PDT. The CTCs derived from a primary tumor circulate in the blood vessels. Since rose bengals (red circles), which are photosensitizers, were injected intravenously prior to CTC-targeting PDT, rose bengals accumulated inside the primary tumor but also the CTCs. When a 473-nm wavelength laser illuminates the blood vessels, the GFP inside the CTCs activates the rose bengal, which produces singlet oxygen. Singlet oxygen induces the destruction of the CTCs inside the blood vessels. **b** Scheme of the in vitro fluidic system mimicking blood circulation coupled with laser irradiation. **c** Proportion of dead cells after irradiation with blue laser light. **d** Differences in cell death between GFP^+^ (green bar) and GFP^−^ (white bar) NCI-H460 cells after up to three rounds of irradiation with the blue laser light. Blue and red signals indicate Hoechst 33342 and propidium iodide (PI) staining, respectively. Y-axis means the ratio of PI-positive cells to total cells. Error bar means standard deviation. *, *P* < 0.05; **, *P* < 0.01; ns, non-specific

In the present study, we demonstrate the clinical benefit of selective CTC elimination by using a technique that we developed previously [10]. We used the original photodynamic therapy (PDT) methodology with stepwise modification to selectively kill CTCs using energy transfer between the green fluorescent protein (GFP) expressed by CTCs and the rose bengal (RB) accumulated in the CTCs (Fig. 1a). To mimic the circulation within the blood vessels in vitro, a piece of tubing was connected to a peristaltic pump. GFP^+^ and GFP^−^ NCI-H460 cells were incubated with RB and were passed through the tubing (Fig. 1b). A greater number of propidium iodide-positive cells (which indicates cell death) was observed among the GFP^+^ NCI-H460 cells than the GFP^−^ NCI-H460 cells. Furthermore, GFP^−^ cells showed lower damage than GFP^+^ cells (Fig. 1c). Moreover, the number of dead cells was significantly higher among GFP^+^ NCI-H460 cells than GFP^−^ NCI-H460 cells (Fig. 1d).

**Fig. 2:**
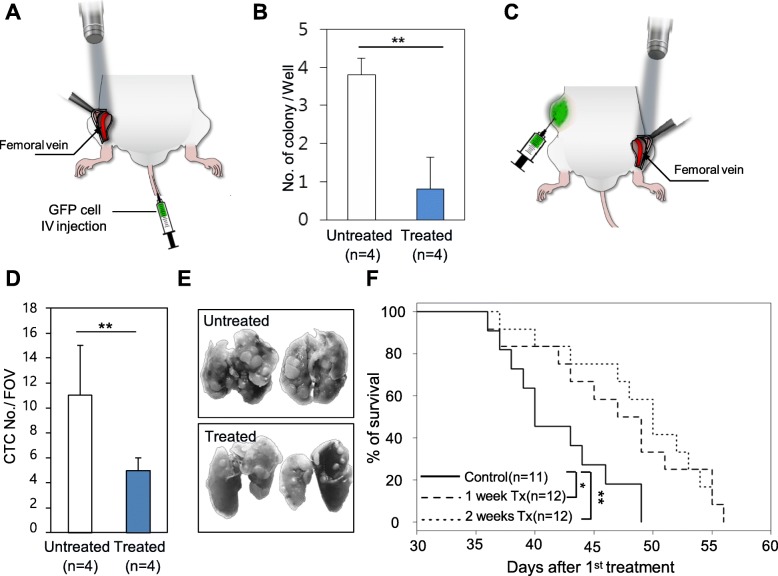
CTC-targeting PDT in the GFP-expressing cancer cell-injected mice model and in a syngeneic mice model implanted with GFP-expressing cancer cells. **a** Irradiation of the mouse femoral vein under the skin flap with a 473-nm wavelength laser after GFP-expressing cancer cell injection via the tail vein. **b** Clonogenic assay using whole blood taken after the experiment. Colonies were stained with Coomassie blue dye, and the number was compared between each group. Error bar means standard deviation. **, *P* < 0.01. **c** Irradiation of the mouse femoral vein under the skin flap with a 473-nm wavelength laser in a syngeneic mouse model with implanted GFP-expressing 4T1 cells. **d** The number of circulating tumor cells in the 2 weeks treatment and untreated mice. Error bar means standard deviation. FOV means the field of view. **, *P* < 0.01. **e** Images of the lungs isolated from mice belonging to the 2 weeks treatment and untreated group. **f** Kaplan–Meier survival curves of the mice in the control and 1 week treatment and 2 weeks treatment groups. *p* values were calculated using the log-rank test between treatment groups and control. *, *P* < 0.05; **, *P* < 0.01

Then, to test the CTC-targeting PDT in vivo, GFP^+^ NCI-H460 cells were incubated with RB and injected into mice via the tail vein. Immediately after, a blue laser was illuminated onto the mouse‘s femoral vein, underneath the skin flap (treated group; Fig. 2a). Because the numbers of CTCs were drastically decreased in the intravenous tumor cell injection model (Additional file 1), whole mouse blood was extracted by cardiac puncture about 15 min after tumor cell injection. In the treated group, the number of CTC colonies were significantly decreased in the clonogenic assay (Fig. 2b and Additional file 2a), and GFP expression from the clones was observed (Additional file 2b); hence, each colony had originated from exogenously injected GFP-expressing cancer cells.

CTC-targeting PDT was also performed in mice with GFP+ metastatic 4T1 cells transplanted into their flanks (Fig. 2c). No changes in primary tumor size (Additional file 3) were observed between treated (irradiated) mice and untreated mice, implying limited effects on GFP^−^ normal cells; however, the numbers of CTCs observed in the fluorescent images were significantly decreased in the treated mice compared to those the untreated mice (Fig. 2d and Additional file 4). In the treated group, the number of lung metastatic nodules in the treated mice was significantly lower compared to that in the untreated group (Fig. 2e). Mice receiving treatment for 1 week showed survival gain compared with untreated mice (*P* = 0.0325) (Fig. 2f). However, the difference was more significant in the mice treated for 2 weeks (*P* = 0.0026). There was no hematologic difference between the untreated group and the 2 weeks treatment group (Additional file 5). Materials and methods are described in Additional file 6.

To prove the benefits of CTC elimination, we developed an energy transfer-based PDT that targets GFP-expressing CTCs. Using this technique, we attempted to eliminate CTCs and optimize conditions to specifically target CTCs, with minimum damage to normal cells. To our knowledge, this is the first experimental study to demonstrate that the direct killing of CTCs extends survival in vivo*.* The present study highlights the concept of energy distinction between normal and cancer cells by using a new factor, i.e., cancer cell-specific fluorescence.

Although this is a preliminary study using the externally fluorescence-labeled cancer cells and the injected mouse models, thus, this strategy is not suitable for in vivo targeting therapeutics of CTC; we reveal that clearance of CTC is associated with the reduction of metastasis and extension of survival. In addition, this experiment directly suggests CTCs are a core seed to be metastasized into secondary organs. Advancements in the field of molecular diagnostics have made it possible to use combinations of fluorescence proteins and photosensitizers or molecular-targeted photosensitizers in diverse biological fields, including cancer stem cell-targeted therapy.

## Additional files

Additional file 1: Changes in colony formation according to the time elapsed since the intravenous injection of NCI-H460 cancer cells**. a** Change in colony formation according to the time elapsed since cancer cell injection. NCI-H460 cells (1 × 10^5^) were intravenously injected into mice and whole blood was collected by cardiac puncture. After lysis of the red blood cells, 200 μL was spread onto a 35-mm dish and incubated for 7 days. The clone numbers were counted using crystal violet staining. The minutes represent the time passed between the cell injection and the blood collection. **b** The change in cancer cell colony number expressed as a graph. ns, not significant; **, *P* < 0.01. (PDF 45 kb)

Additional file 2: Clonogenic assay. **a** Clonogenic assay using whole blood taken after the experiment. **b** The images are close-ups of 1 and 2 indicated in C. GFP signal of each colony was confirmed. (PDF 50 kb)

Additional file 3: Monitoring of primary tumor growth in both the treated and untreated groups. ns, non-specific. (PDF 24 kb)

Additional file 4: Comparison of CTCs and CD45 positive leukocytes in treated and untreated mice. The fluorescent images of CTCs from treated and untreated mice were compared (left panel). Changes in CTC and leukocyte numbers were confirmed by performing EpCAM and CD45 immunostaining, respectively (middle and right panel). (PDF 26 kb)

Additional file 5: Effect of CTC-targeting PDT on hematologic profiles. After 2 weeks of treatment, the blood from four mice was taken and a complete blood count test was performed. The number of white blood cells (WBC), red blood cells (RBC), hemoglobin, and platelets were counted. The results were compared with those from four untreated control mice. ns, non-specific. (PDF 28 kb)

Additional file 6: Supplementary Materials and Methods. (DOCX 18 kb)

## Abbreviations

CTCs: Circulating tumor cells
GFP: Green fluorescent protein
PDT: Photodynamic therapy
RB: Rose bengal
