# Correction to: Selective killing of circulating tumor cells prevents metastasis and extends survival

**DOI:** 10.1186/s13045-018-0688-z

**Published:** 2019-01-09

**Authors:** Yi Rang Kim, Jung Ki Yoo, Chang Wook Jeong, Jin Woo Choi

**Affiliations:** 1 Department of Hematology/Oncology, Yuseong Sun Hospital, Daejeon, 34084 Republic of Korea; 2 Department of Pharmacology, College of Pharmacy, Kyung Hee University, Seoul, 02447 Republic of Korea; 3 Department of Life and Nano-pharmaceutical Sciences, Kyung Hee University, Seoul, 02447 Republic of Korea; 4 Department of Urology, Seoul National University Hospital, Seoul, 03080 Republic of Korea

**Correction to: J Hematol Oncol**

**https://doi.org/10.1186/s13045-018-0658-5**

The original article [1] contains an inadvertent omission in the Funding declaration. The authors would like to amend the Funding statement to the following:

**Funding**

This work was supported by the grants from Kyung Hee University (KHU20170844 for JW Choi) and the National R&D Program for Cancer Control, Ministry of Health and Welfare, Republic of Korea (HA17C0039 for YR Kim, CW Jeong, and JW Choi).
