# Supplementary figures and images for: Selective killing of circulating tumor cells prevents metastasis and extends survival
Source: J Hematol Oncol. 2018 Sep 10;11:114. doi: 10.1186/s13045-018-0658-5 (PMC6131899; doi:10.1186/s13045-018-0658-5)

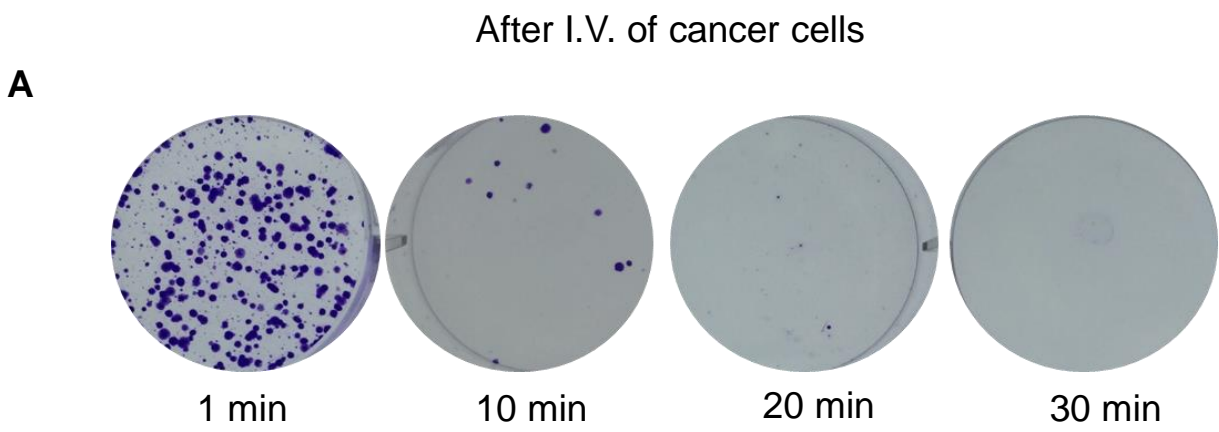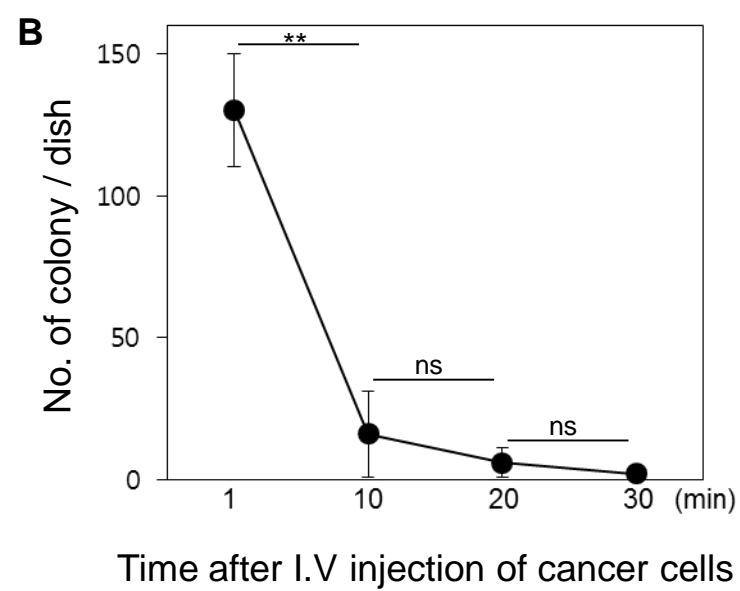

Supplement: Supplementary file 1 — Changes in colony formation according to the time elapsed since the intravenous injection of NCI-H460 cancer cells. a Change in colony formation according to the time elapsed since cancer cell injection. NCI-H460 cells (1 × 10^5^) were intravenously injected into mice and whole blood was collected by cardiac puncture. After lysis of the red blood cells, 200 μL was spread onto a 35-mm dish and incubated for 7 days. The clone numbers were counted using crystal violet staining. The minutes represent the time passed between the cell injection and the blood collection. b The change in cancer cell colony number expressed as a graph. ns, not significant; **, P < 0.01. (PDF 45 kb) [file 13045_2018_658_MOESM1_ESM.pdf]

Additional File 2

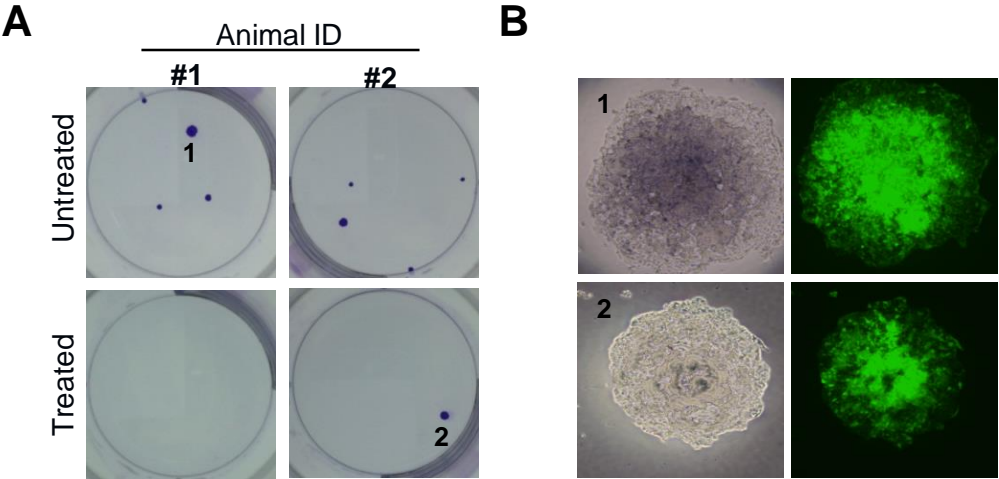

Supplement: Supplementary file 2 — Clonogenic assay. a Clonogenic assay using whole blood taken after the experiment. b The images are close-ups of 1 and 2 indicated in C. GFP signal of each colony was confirmed. (PDF 50 kb) [file 13045_2018_658_MOESM2_ESM.pdf]

Additional File 3

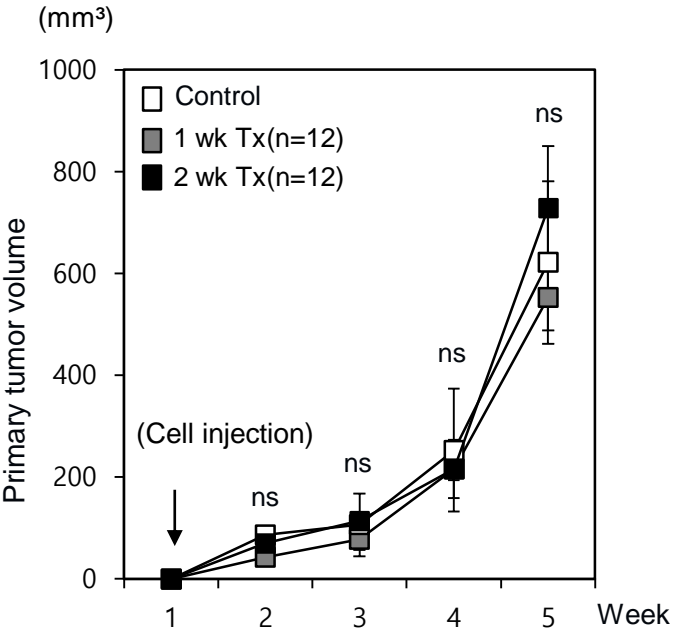

Supplement: Supplementary file 3 — Monitoring of primary tumor growth in both the treated and untreated groups. ns, non-specific. (PDF 24 kb) [file 13045_2018_658_MOESM3_ESM.pdf]

Additional File 4

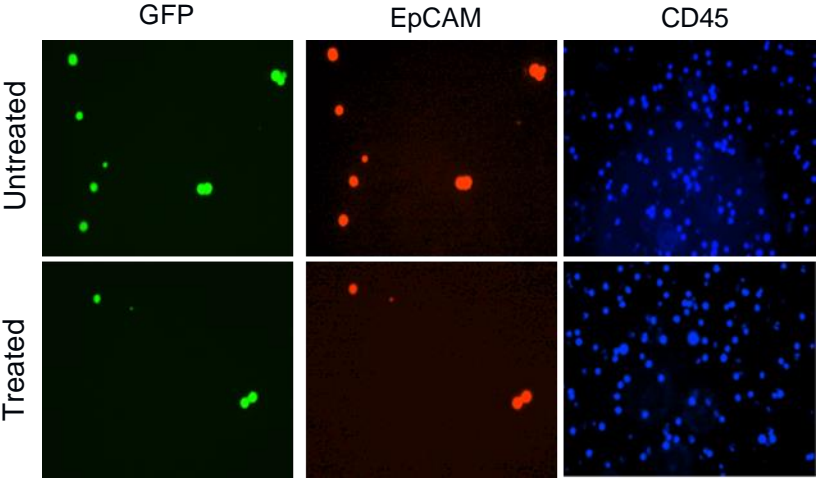

Supplement: Supplementary file 4 — Comparison of CTCs and CD45 positive leukocytes in treated and untreated mice. The fluorescent images of CTCs from treated and untreated mice were compared (left panel). Changes in CTC and leukocyte numbers were confirmed by performing EpCAM and CD45 immunostaining, respectively (middle and right panel). (PDF 26 kb) [file 13045_2018_658_MOESM4_ESM.pdf]

Additional File 5

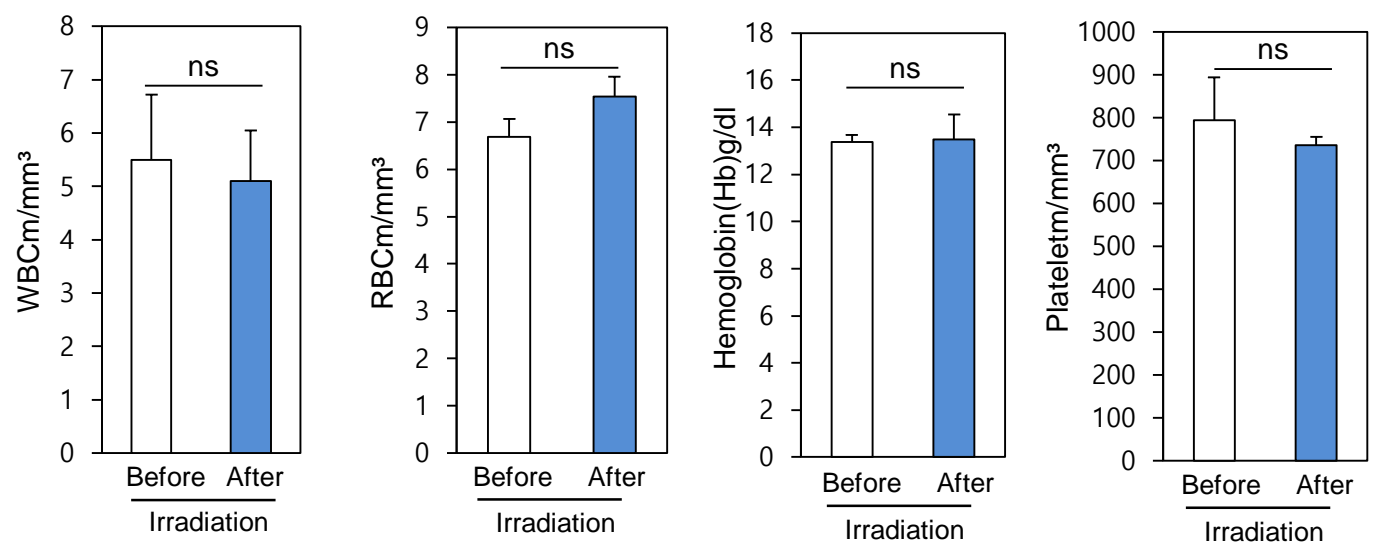

Supplement: Supplementary file 5 — Effect of CTC-targeting PDT on hematologic profiles. After 2 weeks of treatment, the blood from four mice was taken and a complete blood count test was performed. The number of white blood cells (WBC), red blood cells (RBC), hemoglobin, and platelets were counted. The results were compared with those from four untreated control mice. ns, non-specific. (PDF 28 kb) [file 13045_2018_658_MOESM5_ESM.pdf]
