# Supplementary material for: Selective killing of circulating tumor cells prevents metastasis and extends survival
Source: J Hematol Oncol. 2018 Sep 10;11:114. doi: 10.1186/s13045-018-0658-5 (PMC6131899; doi:10.1186/s13045-018-0658-5)
Supplement: Supplementary file 6 — Supplementary Materials and Methods. (DOCX 18 kb) [file 13045_2018_658_MOESM6_ESM.docx]

**Additional Materials and Methods**

**Cell culture**

B16-F10 (*Mus musculus* skin melanoma), NCI-H460 (human non-small cell lung cancer cells) and 4T1 (*Mus musculus* mammary breast cancer) cell lines were purchased from the Korean Cell Line Bank. B16-F10 cells were maintained in Dulbecco’s modified Eagle’s medium (Hyclone); other cells were cultured in Roswell Park Memorial Institute (RPMI)-1640 medium supplemented with 10% fetal bovine serum (FBS; Hyclone) in a humidified atmosphere containing 5% CO_2_ at 37 °C.

***In vitro* blood vessel-mimicking models with blue light irradiation**

B16-F10 and GFP^+^ B16-F10 mouse melanoma cells or NCI-H460 and GFP^+^ NCI-H460 human carcinoma cells were prepared 1 day before the experiment. The cells were incubated with RB (100 µM) for 4 h. Live cells (10^5^ cells/mL) were collected using 20 mL 0.9% saline into a round-bottom tube that was later connected to one side of a peristaltic pump (EP-1 Econo; Bio-Rad) by using a transparent wall tubing (Tygon, E-3603; 4 mm inner diameter × 6 mm outer diameter). An empty tube was prepared to collect the samples at the other end of the pump. The cells were made to flow from one end to the other at a rate of 10 mL/min. Next, a 1 cm region at the end of the tubing was exposed to a 473 nm wavelength blue laser light (45 mW). The cells were flowed through the apparatus up to three times. All procedures were conducted in a dark room to prevent exposure of the cells to light. Finally, the cells were seeded in 12-well plates and stained with Hoechst 33342 and propidium iodide solution, and observed under microscope.

***In vivo* irradiation for eliminating CTCs in CTCs injection model**

Sixteen 6-week-old male nude nu/nu mice were obtained from Orient Bio, Ltd. (Korea). Of these, four mice were intravenously injected with 2 × 10^6^ NCI-H460 cells transfected with the GFP-expressing adenovirus vector, four mice were injected with 2 × 10^6^ NCI-H460 cells pretreated with 100 nM RB, and four mice were injected with 2 × 10^6^ GFP-expressing cells pretreated with 100 nM RB. The remaining four mice were used as negative controls. At 15 min after cell injection, the femoral veins of all the mice were exposed to blue laser light (45 mW), under the skin flap, for 5 min. Next, a clonogenic assay using whole blood taken from the mice post-experiment was performed. Red blood cells were lysed, and the precipitate containing the tumor cells were seeded in 6-well plates. After 2 weeks, the colonies were stained with Coomassie blue dye and the number of colonies from each treatment group was compared. The GFP signal of each colony was confirmed using a florescent microscope.

***In vivo* irradiation for eliminating CTCs in a syngeneic mice model implanted with GFP-expressing cancer cells**

For the survival tests, 44 6-week-old Balb/c mice were purchased from Orient Bio (Seoul, Korea), and 1 x 10^6^ 4T1 cells were transplanted into the flanks of the animals. Primary tumor volumes were measured every week. Three weeks after tumor implantation, RB (30 mg/kg) was intravenously injected 6 hours prior to blue laser (λ = 473 nm) irradiation. The femoral veins were exposed to blue laser light (45 mW), under the skin flap, for 5 min. Twelve mice were irradiated twice a week for 1 week, and another 16 mice were irradiated twice a week for 2 weeks. The remaining 16 mice were used as negative controls. Immediately following two weeks of treatment, 4 mice from the two weeks treatment group and 4 mice from the untreated group were sacrificed. Whole blood was obtained by performing cardiac puncture. Using 200 µL of blood sample, the number of CTCs was counted and confirmed by performing immunostaining with anti-EpCAM antibody (Santacruz Biotechnology, CA. US). White blood cells were counterstained with anti-CD45 antibody (Santacruz Biotechnology, CA. US). Using the rest of the blood sample, the number of white blood cells (WBC), red blood cells (RBC), hemoglobin, and platelets were counted. Survival of the remaining mice was analyzed, and *p* values were calculated using the log-rank test.

**Statistics**

All statistical tests were two-sided, and a P value of less than .05 was considered statistically significant. Student T-tests were used to evaluate statistical significance by comparing differences between treated and untreated group. Survival of the remaining mice was analyzed, and *p* values were calculated using the log-rank test.
